# A case report of the beneficial effects of botulinum toxin type A on Raynaud phenomenon in a patient with lung cancer

**DOI:** 10.1097/MD.0000000000005092

**Published:** 2016-10-07

**Authors:** Lu Wang, Qi-song Lei, Yu-ying Liu, Guan-jie Song, Chun-ling Song

**Affiliations:** a Department of Neurology, Tianjin Baodi Hospital, Tianjin; b Department of Neurology, The Second Hospital of Jilin University, Changchun, China.

**Keywords:** botulinum toxin type A, paraneoplastic, Raynaud phenomenon, visual analogue scale

## Abstract

**Objective:**

Raynaud phenomenon is a vasospastic disorder affecting the hands and feet, and the efficacies of traditional treatments, such as pharmacological therapies and sympathectomy, are not uniform. Patients with paraneoplastic Raynaud phenomenon do not benefit from the traditional treatments. The use of botulinum toxin type A (BTX-A) for Raynaud phenomenon has been reported for several years; however, there are few reports regarding botulinum toxin type A in the treatment of paraneoplastic Raynaud phenomenon. We describe a case report of the beneficial effects of botulinum toxin type A on Raynaud phenomenon in a patient with lung cancer.

**Methods:**

A 63-year-old male complained of pain and discoloration of his fingers and indicated that oral nifedipine and low-dose aspirin were not effective. After approximately 8 months, he was diagnosed with lung cancer. Chemotherapy partially reduced the pain and discoloration of his fingers; however, no significant changes occurred in his fingers after the fourth cycle. We used BTX-A to treat this patient with paraneoplastic RP. A visual analogue scale (VAS) was used to assess the clinical response.

**Results:**

After approximately 2 months, the patient reported relief from pain, stiffness, numbness, and cold sensation. Furthermore, no local or general adverse effects were exhibited by the patient.

**Conclusion:**

This study used botulinum toxin type A for a patient with paraneoplastic Raynaud phenomenon. Botulinum toxin type A significantly improved the patient's clinical symptoms without significant complications. These findings suggest that BTX-A may represent a good option for the treatment of paraneoplastic RP.

## 1 Introduction

Raynaud phenomenon (RP) is characterized by vasospasms of the digital arteries and results in pain, stiffness, numbness, a cold sensation, ischemic ulcers, and even loss of function. The fingers are most commonly affected. The efficacy of existing treatments, such as pharmacologic therapies and sympathectomy, is not uniform. Treatment is ineffective in some patients, particularly those with malignancies. In this article, we report the use of botulinum toxin type A for the treatment of paraneoplastic RP in a patient with lung cancer. According to our results, botulinum toxin type A (BTX-A) for the treatment of paraneoplastic RP is safe and effective.

## 2 Case report

A 63-year-old male complained of an 18-month history of pain and discoloration of his fingers upon exposure to cold. He had no prior history of cardiovascular issues, hypertension, diabetes, hepatitis, trauma, snake-bite, or neuromuscular disorders. The patient did have a history of cervical spondylosis. He indicated no history of drinking and had quit smoking at the age of 60. The patient presented to the rheumatology clinic and was initially diagnosed with RP. The initial pharmacologic treatment for his RP included 15 mg of nifedipine daily and low-dose aspirin. The patient was also treated with prostaglandin E1; however, he could not bear the headaches and had to stop this alternative pharmacological therapy. Unfortunately, no clinical change occurred during the following months.

**Figure 1:**
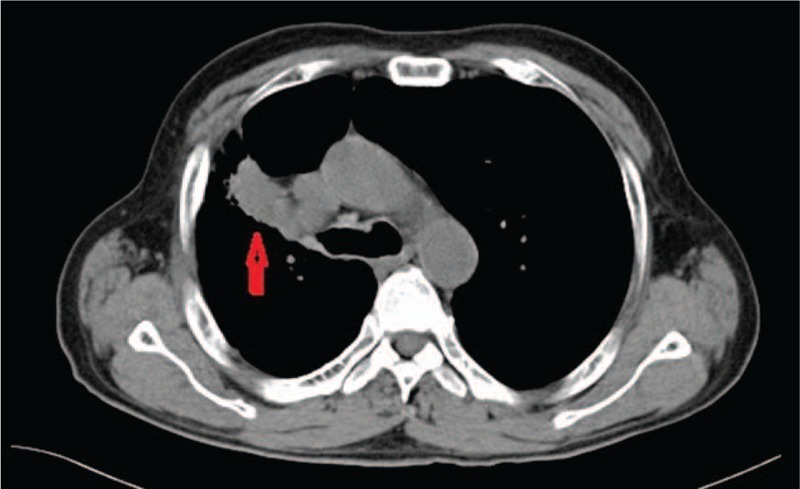
Chest computed tomography (CT) indicates a lesion in the right lung.

**Figure 2:**
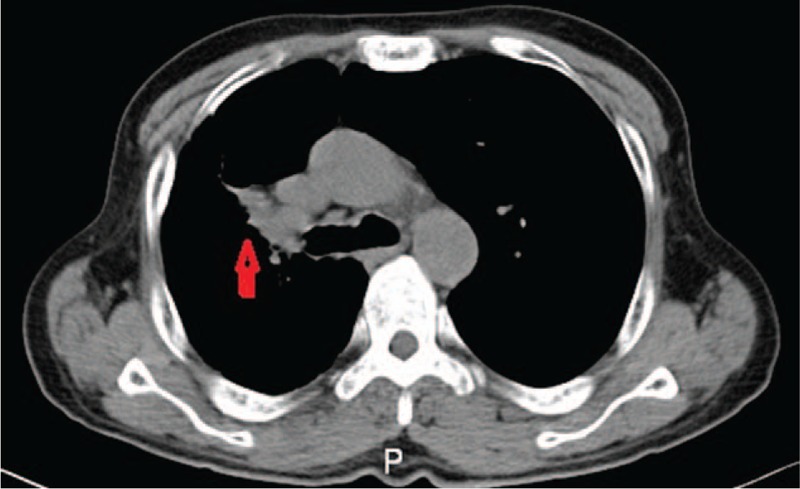
Chest computed tomography (CT) after the fourth cycle of chemotherapy indicates the lesion in the right lung.

Approximately 8 months later, he developed new, severe chest pain. A subsequent chest computed tomography (CT) (Fig. 1) and histologic examination confirmed lung adenocarcinoma. The patient was staged T_2_N_1_M_0_ (stage-IIB) according to the American Joint Committee on Cancer (AJCC) staging manual.^[1]^ The patient was offered chemotherapy based on docetaxel and carboplatin (docetaxel 100 mg and carboplatin 500 mg, 21 day cycle). The patient enjoyed partial reductions in his symptoms after the first and second cycles of chemotherapy for lung cancer. However, no significant changes occurred in his fingers after the fourth cycle. A decrease in the lesion size was identified on the chest CT scan (Fig. 2) after the fourth cycle of chemotherapy. Upon examination, visible pallor, cyanosis, and hyperemia in the fingers were observed. All peripheral pulses were easily felt and intact. The sensory and motor examination of the nervous system was within normal limits, and there was no central cyanosis. No abnormalities were detected regarding antinuclear antibodies, antineutrophil cytoplasmic antibodies, antiphospholipid antibodies, or rheumatoid factor. The total leucocyte count was 6500 cells per microliter, which comprised 59.9% neutrophils and 30.8% lymphocytes. The hemoglobin level was 14.2 g per deciliter. The erythrocyte sedimentation rate was 17 mm in the first hour. Moreover, no abnormalities were detected in the serum protein levels. He was diagnosed with paraneoplastic RP.

**Figure 3:**
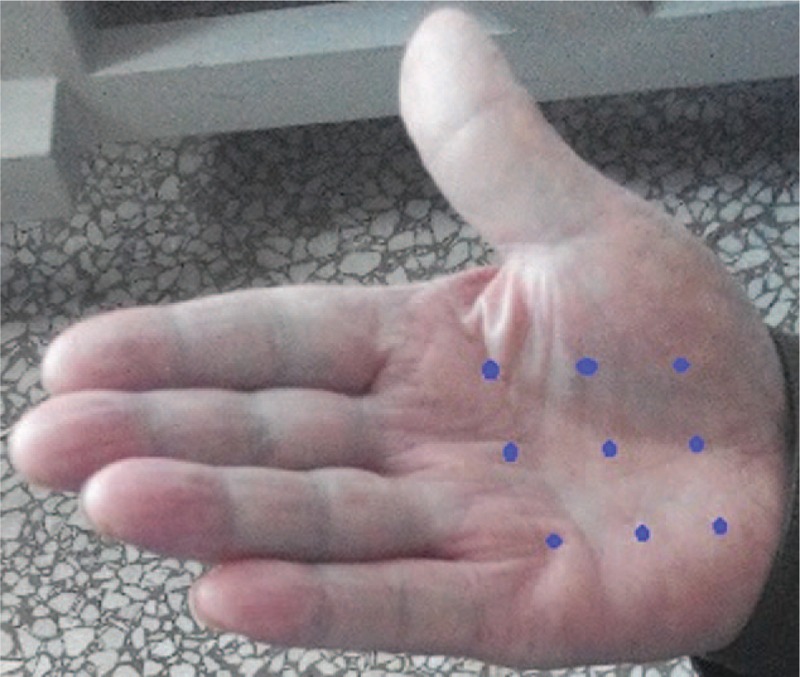
This picture indicates injection points of the palmar.

**Figure 4:**
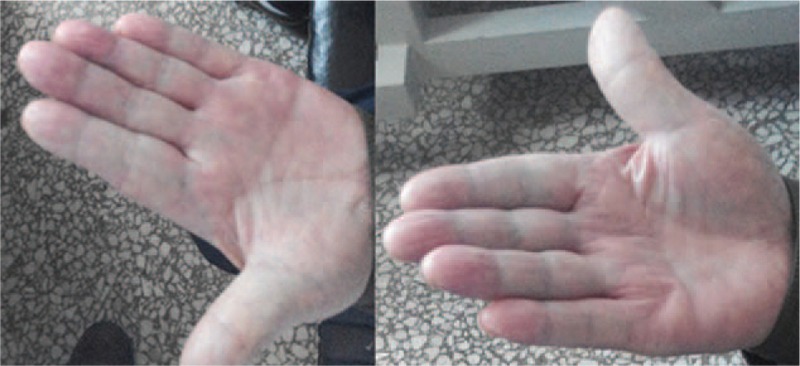
This picture indicates the hands prior to injection.

**Figure 5:**
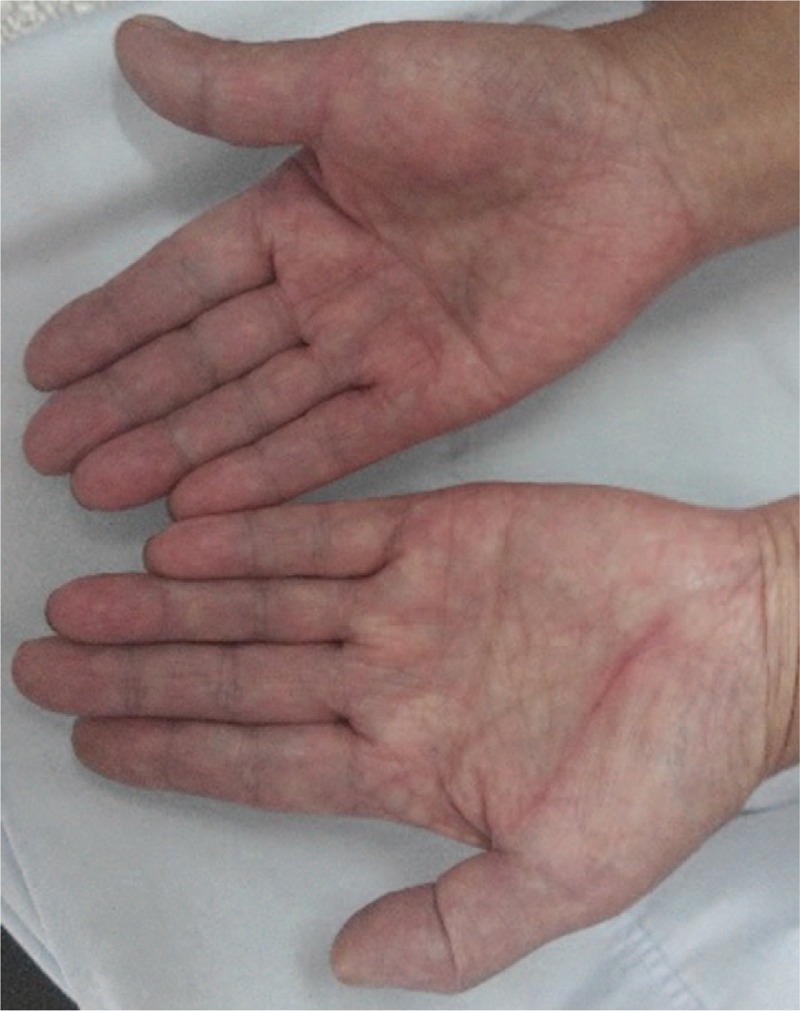
This picture indicates the hands 2 months later after injection.

There have been few reports regarding the use of botulinum toxin type A for the treatment of paraneoplastic RP; thus, we were unsure whether there would be adverse reactions in this case. We informed the patient about the use of BTX-A and obtained consent. Thus, we used BTX-A (BOTOX, Allergen Pharmaceutical Ltd, Westport, Ireland) to treat this patient with paraneoplastic RP. A 100-U vial of BTX-A was diluted with 4 mL 0.9% sodium chloride. Each injection site received 2.5 U. The patient was treated with BTX-A injections into 9 sites on the palm of each hand (Fig. 3). We assessed the clinical response using a visual analogue scale (VAS range 0–10; 0 = normal and 10 = as bad as imaginable) for the clinical symptoms (i.e., pain, stiffness, numbness, and cold sensations). Symptom assessments were performed prior to the injections (Fig. 4) and 2 months later (Fig. 5). The patient reported relief from pain (VAS: pre: 9; post: 1), stiffness (VAS: pre: 8; post: 2), numbness (VAS: pre: 7; post: 4), and cold sensation (VAS: pre: 8; post: 1). The effects of BTX-A persisted for least 6 months after the injections. Neither local or general adverse effects were observed in the patient.

## 3 Discussion

RP results from vasospastic disorders of the digital arteries and leads to pain, stiffness, numbness, cold sensations, and ulcers and even affects daily activities. The incidence of RP is approximately 3% to 5% among the general population.^[2]^ RP can be classified into primary and secondary RP. Secondary RP can be associated with various autoimmune diseases, including tissue disorders (e.g., rheumatoid arthritis, scleroderma, Sjogren syndrome, systemic lupus erythematosus, dermatomyositis, and mixed connective tissue disease), vessel disorders (e.g., Buerger disease, atherosclerosis, and Takayasu arteritis), histories of specific drugs, hepatitis, snake-bite, cryoglobulinemia, and malignancy. The RP in our patient was associated with lung cancer and was diagnosed as paraneoplastic RP. The pathophysiology of RP is multifactorial and includes vascular, intravascular, and neural abnormalities.^[3]^ The plausible mechanism of paraneoplastic RP includes a hypersensitive reaction to the antigens of tumor cells and vascular endothelium.^[4]^ Hsu et al^[5]^ reviewed in their article that there are various potential pathogenic mechanisms, such as tumor invasion of sympathetic nerves, hypercoagulability, hyperviscosity, vasoactive tumor-secreted substances, generalized vasospasm, and spontaneous platelet aggregation.

Pharmacologic management including calcium channel blockers and topical nitrates has been administered to patients with RP. However, some patients, particularly those with paraneoplastic RP, are recalcitrant to such treatments. Occasionally, treatment of the malignancy can alleviate paraneoplastic RP.^[6]^ BTX-A has wide therapeutic applications, including the treatment of blepharospasm, facial spasms, spasms of the extremities, cervical dystonia, axial hyperhidrosis, and migraines.^[3]^ Sycha et al^[7]^ first reported the use of BTX-A for RP in a cases series with 2 patients, and their data suggested a beneficial effect. Since then, several studies have assessed the effects of BTX-A for the treatment of RP. All of those studies reported promising clinical results. However, BTX-A is not suitable for patients with myasthenia gravis or Lambert–Eaton myasthenia syndrome.

Our study revealed a beneficial effect of BTX-A in a patient with paraneoplastic RP. Thus far, there have been no obvious adverse events, such as allergic episodes, weakness, or effects on lung cancer. One mechanism of action of BTX-A may affect vascular smooth muscles by blocking the transmission of norepinephrine vesicles and preventing sympathetic vasoconstriction. Moreover, BTX-A may specifically block the recruitment of α_2_-adrenoreceptors, which can lead to a reduction in vasoconstriction and pain.^[8,9]^ Another mechanism has been inferred from animal cutaneous flap models and suggests that BTX-A increases the blood flow and inhibits spasms and vascular contractions.^[10]^ The potential mechanism of BTX-A in the treatment of paraneoplastic RP is that BTX-A may inhibit generalized vasospasm and subsequently cause vasodilation, in combination with the function of increasing the blood flow, which may be helpful in reducing spontaneous platelet aggregation and hyperviscosity.

BTX-A has been used to relieve the symptoms of vasospasm in patients with RP without significant adverse events. Additional investigations are needed to understand the mechanism of action and appropriate dose and dose frequency.

## 4 Conclusion

BTX-A may be an effective treatment for paraneoplastic RP that is not associated with significant complications.

## 5 Ethical approval

This study was approved by the institutional Review Board or Ethics Committee of the second hospital of Jilin University, Changchun, China. The authors affirm that the patient depicted in the images and via other personal information provided written informed consent for the dissemination of the data for educational and research purposes.
